# The Role of Mobile Health Technology in Perioperative Spinal Care: A Systematic Scoping Review and Narrative Synthesis

**DOI:** 10.7759/cureus.54254

**Published:** 2024-02-15

**Authors:** Jafar Hayat, Mohammed Ramadhan, Ahmed R Gonnah, Alwaleed Alfadhli, Abdulrahman O Al-Naseem

**Affiliations:** 1 General Surgery, Sheikh Jaber Al-Ahmad Al-Sabah Hospital, Kuwait City, KWT; 2 Medicine, Ministry of Health, Kuwait, Hawally, KWT; 3 School of Medical Sciences, The University of Manchester, Manchester, GBR; 4 Medicine, Imperial College Healthcare NHS Trust, London, GBR; 5 Faculty of Medicine, Royal College of Surgeons in Ireland, Dublin, IRL; 6 Division of Surgery & Interventional Science, University College London, London, GBR

**Keywords:** ortho surgery, spinal column, spine injury, spine rehab, mobile technology

## Abstract

Smartphone applications play a crucial role in contemporary healthcare by aiming to enhance patient care through technology. Mobile health (mHealth) applications have proven to have transformative potential in enhancing patients' outcomes in candidates undergoing orthopedic and spinal surgery. In the context of the pervasive use of smartphones and the exponential growth of mHealth apps, totaling over 99,000 in 2021, the applications had a significant impact on lifestyle management, supporting initiatives like smoking cessation with motivational reminders and progress tracking. Patient compliance is significantly enhanced, reducing surgery cancellations and improving outcomes through effective adherence to pre-operative treatments and instructions. Physiotherapy receives a substantial boost as mHealth facilitates video-guided exercises, potentially improving compliance and treatment outcomes. Data collection takes on innovative dimensions, with mHealth apps capturing post-operative metrics like physical activity, offering valuable insights into patient recovery trends. Remote care is streamlined through features like photo uploads and direct messaging, proving especially beneficial in times of crises such as the COVID-19 pandemic. Despite these merits, challenges emerge, including issues related to technological literacy, potential discrimination due to paywalls, and concerns about patient data confidentiality. Overcoming these challenges requires standardized approaches, legislative measures, and ongoing research to refine and optimize mHealth applications for diverse healthcare settings.

## Introduction and background

Smartphones have become an omnipresent tool in the majority of people's lives since their inception in the early 21st century. With an exponential rate of growth in the past decade, and there being over 99,000 mobile health (mHealth) applications as of 2021 [1], it is only rational that there be an assessment and understanding of the medical, legal, and social implications of their use in healthcare [2]. These applications exist to facilitate many aspects of medical practice including health record maintenance and access, communication and consulting, reference and information gathering, patient management and monitoring, as well as clinical decision-making, medical education and training. This positively impacts patients’ outcomes, but also improves healthcare services efficiency and reduces their costs over an extended period of time [3-11]. The role of mHealth apps also extends into the entirety of the perioperative landscape, including the process before, during, and after surgery [12]. These applications also help enhance the quality of perioperative outcomes such as pain, wound healing, and recovery, tackling factors that lead to increased delays [13, 14], morbidity, and mortality in surgery [15, 16]. There are a multitude of spine conditions including spinal fractures, scoliosis, and degenerative cervical myelopathy that require extended post-operative follow-up which can be facilitated by mHealth [17, 18]. This review aims to highlight the benefits and the potential limitations of mobile health applications, based on the current literature, in managing patients undergoing orthopedic and spinal surgery.

## Review

Methods

Search Strategy

We systematically searched the databases PubMed, EMBASE, and Web of Science under PRISMA guidelines looking for observational studies that assess the role of mHealth applications on perioperative spine patients. The search terms used included: “mobile“ and “mHealth“ and “Spine” and “Phone” and “App” and “Application” and “Tele” and “Telephone”. The review period was restricted from January 1, 1930, to June 30, 2023.

Eligibility criteria and study selection

Inclusion Criteria

Inclusion criteria were all levels of scientific evidence, any treatment option, human studies, both genders and any age group.

Exclusion Criteria

The following criteria were used for exclusion: articles of unrelated diagnosis, articles available in abstract form only and non-English articles.

Data extraction

Each abstract was screened for possible inclusion by two reviewers (JH, AN) independently. If a consensus was not reached, a third author (MR) was consulted. Two authors (AF, AN) performed data extraction.

Outcomes of interest

Primary Outcomes

Studies were required to have used mHealth applications and record outcomes in their respective observational cohorts. Ideally, studies used a validated metric through the use of PROMs (patient-reported outcome measures).

Secondary Outcomes

We also extracted data on the reported limitations of the available studies, focusing on elements that may make results challenging to repeat.

Intervention

The use of mHealth applications in any form on the outcomes of spine patients perioperatively.

Comparator/control

No comparison or control group was required for inclusion in this review.

Results

**Figure 1:**
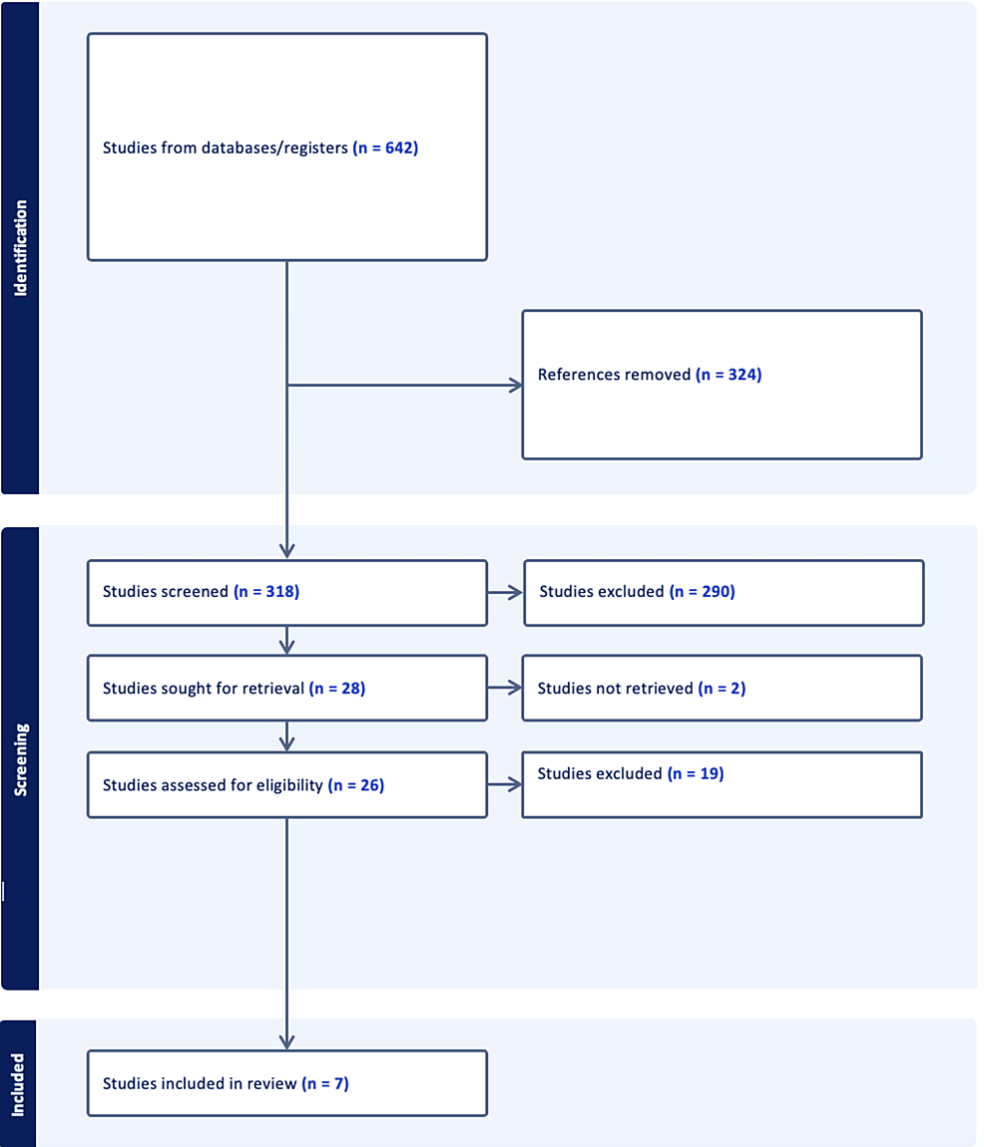
Screening and extraction process of the review

The literature search identified 642 studies and after a thorough screening of the retrieved articles, a total of seven studies met the eligibility criteria (Figure 1).

Numerous studies probing the role of mHealth in perioperative spine patients unveil valuable insights (Table 1). Ponder et al. revealed the beneficial impact of mHealth on pre- and post-operative patient adherence, leading to a reduction in last-minute surgery cancellations due to a decreased likelihood of misunderstanding instructions on patients undergoing spine surgery [19].

**Table 1:** Table of observational studies in available literature of mHealth on spine patients

Number	Title of Study	Author	Year	Type of Study	Primary Outcome	Patients	mHealth Instrument	Operative Status	Reported Benefits	Limitations
1	Use of a Smartphone Application for Spine Surgery Improves Patient Adherence with Preoperative Instructions and Decreases Last-minute Surgery Cancellations	Stewart et al.	2019	Prospective Cohort	Demonstrating the link between mHealth and compliance to pre-operative instructions and reduction of surgical cancellations on spinal patients.	85	FavorHealth Application	Pre-operative	Increased adherence (No canceled surgeries versus 5 canceled surgeries for non-application users)	Limitations of this study include a relatively small patient cohort and lack of randomization. Moreover, patients with smartphones or tablets who are facile with apps are generally younger and more likely to retain information when compared to older patients.
2	A Smartphone App With a Digital Care Pathway for Patients Undergoing Spine Surgery: Development and Feasibility Study	Ponder et al.	2020	Prospective Cohort	A feasibility study of a smartphone application called ManageMySurgery (MMS) to assess the outcomes of patients undergoing elective spine surgery.	47	ManageMySurgery (MMS)	Perioperative	Of the 24 patients who completed the MMS survey, 21 (88%) said it was helpful during preparation for their procedure, 16 (67%) said it was helpful during the postoperative period, and 23 (96%) said that they would recommend MMS to a friend or family member.	Participation bias may have influenced the feasibility study, and the use of descriptive statistics may devalue certain data; alongside the application being in the early stages of development.
3	Patients undergoing surgery for lumbar degenerative spinal disorders favor smartphone-based objective self-assessment over paper-based patient-reported outcome measures	Sosnova et al.	2020	Prospective Cohort	To assess patients' preference of an objective smartphone-based outcome measures compared to conventional paper-based methods for lumbar degenerative spinal disorder patients.	49	6-minute Walking Test application (6WT-app)	Perioperative	The majority of patients considered the 6WT-app a suitable instrument and preferred it over traditional methods. There was good agreement that the 6WT-app detects changes in physical performance (8.0, IQR 4.0). 78% of patients considered the 6WT superior in detecting differences in symptoms. Eighty-two percent of patients indicated their preference to use a smartphone app for the assessment and monitoring of their spine-related symptoms in the future.	Data is limited by the number of individuals in the study; may not represent the total population. Data is influenced by patient intelligence and access to software.
4	Patient Participation With a Mobile Phone Application for Objective Activity Assessment Before and After Spinal Fusion	Sprau et al.	2020	Retrospective Cohort	Data collecting and analyzing patient demographics who participated in the use of smartphone app "QS Access" as a method to assess patients' functional statuses surrounding spinal fusion.	41	Quantified Self Labs's "QS Access" and Apple iPhone Health Data	Perioperative	Improved reporting of patient data in comparison to traditional means; improved convenience; well received by patients.	15 patients were not able to communicate in English from the sample size, and not all data acquired was usable by the team.
5	Smartphone-based real-life activity data for physical performance outcome in comparison to conventional subjective and objective outcome measures after degenerative lumbar spine surgery	Voglis et al.	2022	Prospective Cohort	Prospective observational study of DLD patients. Objective functional capacity and subjective outcomes were measured using 6WT and PROMs. Real-life physical performance data were acquired retrospectively using Apple iPhone Health data and compared against objective capacity and subjective outcomes.	8	6-minute Walking Test application (6WT-app) and Apple iPhone Health Data	Perioperative	At the 6- and 12-week follow-up significant improvements were observed in all PROMs. Using the 6WT as a smartphone app-based functional test they were able to show that the improvement in PROMs is accompanied by an increase in physical capacity, indicating the gradual decrease in objective functional impairment three months after surgery. This is also aided by improved reporting of data compared to traditional means.	Small sample size and the calculated pre- and postoperative trends are based on repeated measures (daily mile counts) and therefore struggle to be represented by a small cohort size. Secondly, ideally, they would like to standardize results by comparison to healthy population values.
6	Smartphone GPS signatures of patients undergoing spine surgery correlate with mobility and current gold standard outcome measures	Boaro et al.	2021	Retrospective Cohort	Measuring patient PROMs (VAS/ODI/PROMIS) through eight daily mobility features of 39 spine surgery patients perioperatively.	39	Smartphone GPS Data	Post-operative	The results of this study demonstrated the ability of smartphone-based GPS mobility features to accurately characterize peri-operative mobility trends in patients undergoing surgery for spine-related diseases and the presence of significant correlation with gold-standard patient-reported outcome measures.	First, patients who did not own a smartphone were a priori excluded, potentially inserting a selection bias towards specific groups, for example, older patients. Similarly, non-English speaking patients were excluded as well. In terms of spine diseases and type of surgical intervention, the population was quite heterogeneous, and our analysis did not take into consideration disease or treatment-specific features.
7	Effectiveness of App-Delivered, Tailored Self-management Support for Adults With Lower Back Pain–Related Disability A SELFBACK Randomized Clinical Trial	Sandal et al.	2021	Randomized Controlled Trial	To investigate the effectiveness of selfBACK, an evidence-based, individually tailored self-management support system delivered through an app as an adjunct to usual care for adults with LBP-related disability.	232	The selfBACK application	Non-Operative	Among adults who sought care for LBP, those who were randomized to receive selfBACK, an evidence-based and individually tailored self-management support system delivered through an AI-based app as an adjunct to usual care, showed reduced pain-related disability at 3 months compared with those who were randomized to receive usual care alone.	First, the participants were not blinded. Second, healthcare use was not monitored during the follow-up. Third, the step-detecting wristband worn by participants in the intervention group may have introduced an additional benefit that is independent of using the selfBACK app. Fourth, the per-protocol analyses could be biased if participants who engaged with the app during the follow-up period had a different prognosis from those who had little app usage.

Sosnova et al. showcased the superiority of mHealth applications over paper-based methods in terms of adherence and convenience, resulting in heightened patient compliance in lumbar degenerative spinal disorder patients [20]. Sprau et al. underscored the positive reception of smartphone-based applications by spinal fusion patients, suggesting their potential as objective operative metrics [21]. Sandal et al. indicated that AI-based mHealth systems, as an adjunct to usual care, mitigated lower back pain-related disability at three months, with this improvement sustained at nine months for lower back pain patients [22].

Voglis et al. demonstrated postoperative improvement in daily physical performance using mHealth applications for degenerative lumbar disease patients, acknowledging challenges in accurate activity monitoring [23]. Boaro et al. showcased smartphone-derived GPS features accurately characterizing perioperative mobility trends and improved data collection capabilities post-operatively using mHealth applications for spine surgery patients [24]. Stewart et al. observed increased adherence among patients using mHealth applications in following pre-operative instructions, correlating with a decrease in last-minute surgery cancellations for spine surgery patients [25].

Discussion and literature review

Advantages of mHealth

**Figure 2:**
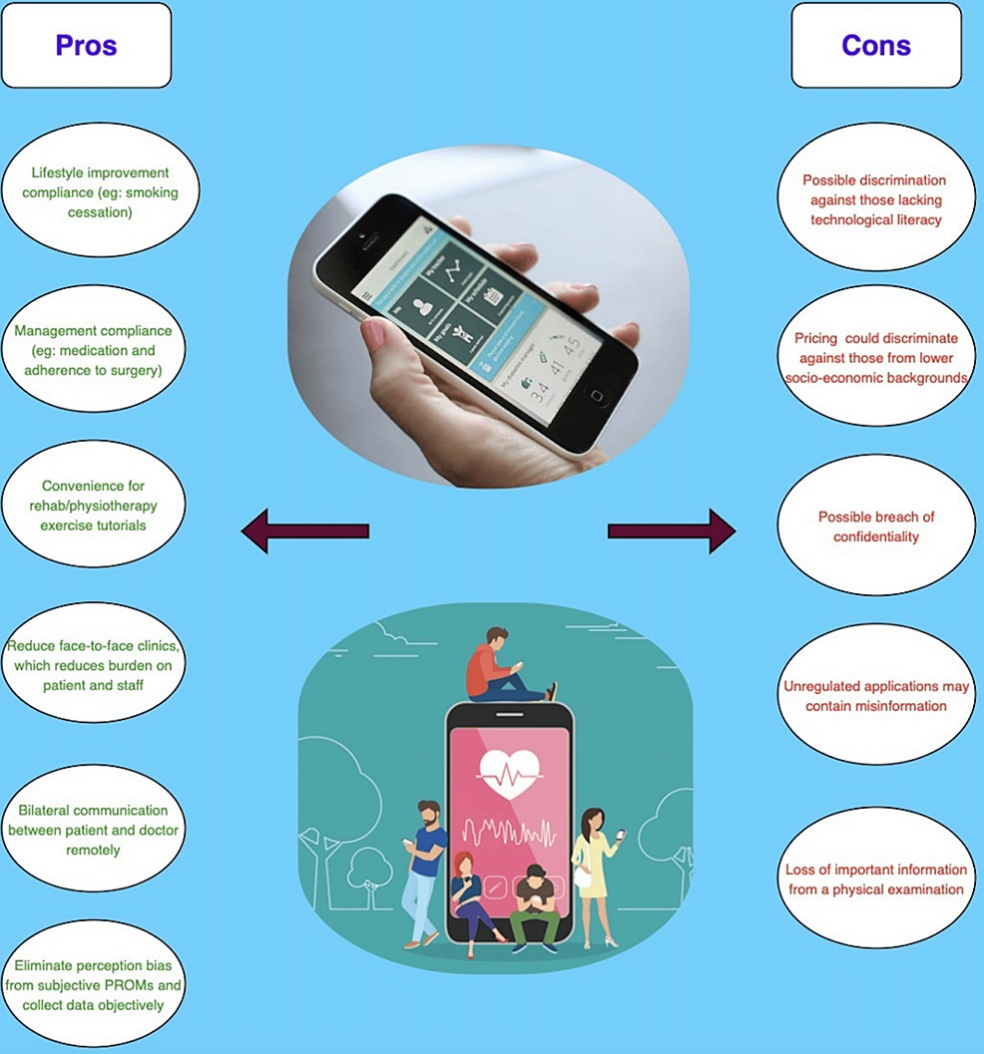
Summary list of pros and cons of implementing mHealth applications This figure is the original work of the authors

Mobile health (mHealth) has proven to be highly beneficial through various approaches in enhancing the health status of patients and improving clinical outcomes, as listed in Figure 2 below. The multifaceted advantages offered by mHealth highlight its transformative impact on healthcare delivery. Below are detailed insights into the diverse modalities by which mHealth contributes to positive healthcare outcomes:

Lifestyle management

Improving patient lifestyle is imperative in improving health outcomes. One example is via smoking cessation support, with reminders being sent to patients to support motivation, in addition to a platform for tracking their progress in quitting [26-27]. Patients smoking prior to surgery could lead to being unfit for surgery, causing delays and possible excess burden on the healthcare system. Additionally, smoking has been associated with a significant reduction in wound healing which could delay progress and worsen patients’ outcomes. Spinal surgery patients may particularly benefit from this, with specific conditions like scoliosis being managed by sending reminders for posture improvement as part of management. Interventions using SMS messages have also been used to influence BMI, associated with osteoarthritis and the need for surgery, which, if reduced, will again reduce the burden on both patients and the healthcare system [27].

Compliance

Adherence to pre-operative medical regimen and instructions could dictate whether a surgery can proceed. A study investigating the effect of mHealth apps on adherence to treatment and instructions involved a cohort of 85 individuals using a mobile application and 89 individuals not using an application, investigating their adherence to medication. All application users were found to be compliant, with five recipients from the control group lost to follow-up. Non-adherence led to the cancellation of surgery, and hence worsening of outcomes for patients, as well as increased cost on the healthcare system [28]. Larger scale trials are required to further investigate this effect. Orthopedic patients commonly receive extensive analgesic regimens post-operatively, with some struggling to comprehend the instructions of use effectively [29]. mHealth applications have facilitated the provision of an easy-to-access platform to provide them with reminders such as detailing when to take vitamin D and bisphosphonates. Patient satisfaction has been increasing with the evolution of mobile health applications where spinal surgery recipients provided a 96% approval rate when rating the benefits of a mobile health application during the course of their treatment [19], with another application receiving an approval from 40 out of 49 patients [20].

Physiotherapy

Physiotherapy is a vital stage of recovery in patients undergoing spinal and orthopedic surgery. Physical therapy may last several months due to fragility fractures [30], and with most of the patients’ demographic being frail, optimizing treatment is imperative. Mobile health applications have shown efficacy in improving patient compliance with physiotherapy [31]. It provides a platform with detailed videos of the exercise programs, which saves the time, effort and costs of traveling to see a physiotherapist. This also improves compliance and may potentially enhance their clinical and health-related quality of life outcomes. The use of mobile health applications to treat back pain was investigated in trials and has displayed a short-term improvement at three months, when compared to conventional treatment without an application [22,32]. However, further larger-scale trials with increased power are required to establish statistical significance. Additionally, there has been evidence of short-term improvement for hip and knee osteoarthritis patients [33]. Moreover, patients with spinal cord injury benefit as they undergo physiotherapy to preserve and improve their neuromuscular function [34-35].

Data collection

Mobile health applications bring an innovative approach in gauging patient recovery post-operatively. Apple's ‘Health’ application on iOS was studied in 2020, collecting metrics within the smartphone of 41 patients who underwent trans-foraminal lumbar interbody fusion (TLIF). The metrics included total steps and distance traveled [21,36]. Patient smartphones were also used in another study conducted in 2022, to measure the 6-minute walking test (6-MWT), as well as survey patient-reported outcomes (PROMs) after degenerative lumbar disease surgery [23]. Collecting metrics of physical activity has been found effective in monitoring recovery trends of patients by assessing the post-operative progress, which is displayed in terms of an increase in daily steps or distance traveled [23]. Another study discussed the use of global positioning system data for an array of spinal conditions including herniated discs, spondylosis, and central canal stenosis, to monitor patients’ activity, post-operatively [24]. This is increasingly beneficial for patients and healthcare professionals to monitor progress and assess procedural success and room for improving management.

Remote care

Smartphone applications could implement a platform to upload progress photos of patients when required, assisting remote monitoring post-operatively [37]. Regular progress pictures can assist scoliosis patients monitor their posture, which could be processed in an application to track progression over time and assess for improvement, to guide future direction of management. Applications may also implement a direct messaging system between patient and clinicians, further strengthening means of communication. These options proved significantly advantageous in the context of COVID-19 [37]. The benefits may be similar to those noted with remote rehabilitation/physiotherapy: saving clinic slots for more acute cases, and reducing patient effort and costs in attending follow-up appointments. Disadvantages that arise from the use of mobile health applications include the difficulty of relying on photos for diagnosis, the loss of physical examinations when using remote care, discriminating patients who have no access to smartphones and the overall market regulation.

Obstacles in the implementation of mHealth

While mHealth applications can be advantageous, there exists a handful of disadvantages that may compromise the quality of care provided, as detailed in Figure 2. The introduction of novel products inevitably brings forth unique challenges that may impede their expansion, utilization, and overall effectiveness. In the context of healthcare services, the development and adoption of applications present various obstacles. Mobile health applications rely on technological literacy. Certain demographics may lack familiarity with the use of the technology, particularly those who may be more elderly which is the same demographic more likely to be vulnerable to spine diseases [20, 32, 33, 37]. Developing applications with a user-friendly interface could help resolve this issue, with some studies attempting to word questionnaires on the application to match sixth-grade reading levels [19]. Additionally, discrimination amongst patients based on financial background and access to healthcare is an issue, because mHealth applications vary in pricing, and some patients would have no access to care. This could leave patients of lower socio-economic status lacking access [20, 32, 38]. It is imperative to intervene and make the applications accessible to people of all ages and socio-economic statuses to ensure that there exists no discrimination against different population demographics.

Confidentiality stands as a paramount principle in the medical profession, and any compromise in patients' information integrity poses a potential threat. Such breaches can impede the adoption of mHealth applications as a standard of care [37]. To address this concern, it is crucial to implement robust encryption and security measures for application data. However, the current market saturation of mHealth applications presents a dual challenge. Firstly, the sheer number of available options may confuse patients, making it difficult to discern quality products from subpar ones. Consequently, this dilutes the capability of high-quality applications to stand out [24]. Secondly, the absence of stringent regulations in the market heightens the risk of misinformation, potentially compromising patient safety [27, 38]. Furthermore, it is essential to acknowledge the limitations of mHealth applications. While they offer a valuable adjunct to healthcare delivery, they cannot fully replace the benefits derived from face-to-face physical examinations - a cornerstone of clinical assessments. Therefore, the optimal approach involves considering mHealth applications as complementary tools that enhance the capabilities of healthcare professionals rather than as complete replacements.

Costs are a big limitation to the implementation of mHealth applications. Even for relatively modest health applications, the investment required can exceed $150,000, dissuading financially constrained practices from considering their implementation [39]. While the long-term benefits may be promising, these initial costs pose a formidable barrier to entry. The ongoing maintenance of healthcare applications also poses financial burdens, particularly for smaller providers. Approximately 90% of software life costs are attributed to the maintenance phase [40]. Addressing this challenge could involve leveraging standardized application builders or no-code software development platforms, which could significantly reduce costs at the expense of relinquishing some control over the frontend of the platform [41, 42].

Another critical concern revolves around patient privacy and ethical considerations. The collection of data, such as patient geolocation and actigraphy, often occurs without participants' full awareness. This raises issues related to informed consent, transparency, and voluntary participation, along with safeguarding data in the event of breaches [43]. While this is a broader issue applicable to all applications, specific legislation focusing on health data privacy, security, and breach notification may be necessary [44,45]. The integration of mobile health applications into healthcare systems faces technical and individual barriers. Challenges include the lack of existing technology infrastructure, interoperability issues, and concerns regarding user-friendliness [46]. Overcoming technological illiteracy and providing adequate training for both staff and patients are additional hurdles [47, 48]. Notably, the elderly population may face challenges due to a lack of familiarity with mobile applications, emphasizing the need for simplified interfaces and literacy promotion [49].

While these obstacles warrant careful consideration by healthcare providers, maintaining a positive outlook on the potential utility of mHealth applications in the future is rational. Addressing these challenges through thoughtful solutions, such as standardized development platforms, legislative measures, and user-friendly interfaces, can pave the way for the effective integration of these applications into healthcare services.

Future considerations

Envisioning a multitude of forthcoming advancements, the continual development of technology and its myriad innovations stands out. In the context of mHealth applications, anticipations of improved cost efficiency [50] and enhanced user-friendliness [51] are on the horizon. As technological literacy grows and the ubiquity of devices facilitating access to these applications increases [52], the prospect of heightened reach and accessibility to the population becomes apparent. Areas of development encompass enhanced accessibility for the visually impaired [53] and cognitively impaired [54], coupled with the standardization of application templates for cost-effectiveness, particularly beneficial for smaller organizations. Standardization not only bolsters security against hijacking but also streamlines overall application usage [55].

Despite existing imperfections, the substantial potential of these applications in advancing the healthcare industry endures. Opinions from literature reviews strongly indicate the resilience and probable ascent of mHealth applications as a major healthcare factor in the upcoming decades [56]. Future research should hone in on addressing application weaknesses and offering pragmatic solutions. Notably, the identified flaw of low sample sizes in existing studies is anticipated to ameliorate as organizations embracing mHealth applications continue their adoption [57].

## Conclusions

The efficacy of mHealth apps in supporting the management of orthopedic and spinal surgery patients is evident. These applications offer a myriad of benefits, ranging from enhancing convenience for both patients and medical teams to mitigating biases in patient data collection, thereby contributing to future research endeavors. However, it is crucial to acknowledge and address potential drawbacks. One significant concern revolves around health inequities, particularly for technologically illiterate or lower socio-economic populations. Ensuring the accessibility and user-friendliness of these applications for diverse demographics is essential to prevent disparities in healthcare access. Additionally, addressing security concerns, such as the potential for breaches in confidential patient data, is paramount for maintaining trust in these digital healthcare tools. Moreover, the absence of a physical element in healthcare delivery through mHealth apps poses a challenge. The value of in-person interactions and physical examinations cannot be entirely replaced, emphasizing the need for a balanced approach that integrates these applications without compromising the essential aspects of traditional healthcare. To enhance the overall efficacy of mHealth applications, further research is recommended. This research should focus on improving patient accessibility, fortifying security measures, and expanding the reach of these applications. By addressing these areas, the potential benefits of mHealth apps can be maximized while minimizing potential drawbacks.
